# Bezielle Selectively Targets Mitochondria of Cancer Cells to Inhibit Glycolysis and OXPHOS

**DOI:** 10.1371/journal.pone.0030300

**Published:** 2012-02-03

**Authors:** Vivian Chen, Richard E. Staub, Sylvia Fong, Mary Tagliaferri, Isaac Cohen, Emma Shtivelman

**Affiliations:** BioNovo, Inc., Emeryville, California, United States of America; University Health Network, Canada

## Abstract

Bezielle (BZL101) is a candidate oral drug that has shown promising efficacy and excellent safety in the early phase clinical trials for advanced breast cancer. Bezielle is an aqueous extract from the herb *Scutellaria barbata*. We have reported previously that Bezielle was selectively cytotoxic to cancer cells while sparing non-transformed cells. In tumor, but not in non-transformed cells, Bezielle induced generation of ROS and severe DNA damage followed by hyperactivation of PARP, depletion of the cellular ATP and NAD, and inhibition of glycolysis. We show here that tumor cells' mitochondria are the primary source of reactive oxygen species induced by Bezielle. Treatment with Bezielle induces progressively higher levels of mitochondrial superoxide as well as peroxide-type ROS. Inhibition of mitochondrial respiration prevents generation of both types of ROS and protects cells from Bezielle-induced death. In addition to glycolysis, Bezielle inhibits oxidative phosphorylation in tumor cells and depletes mitochondrial reserve capacity depriving cells of the ability to produce ATP. Tumor cells lacking functional mitochondria maintain glycolytic activity in presence of Bezielle thus supporting the hypothesis that mitochondria are the primary target of Bezielle. The metabolic effects of Bezielle towards normal cells are not significant, in agreement with the low levels of oxidative damage that Bezielle inflicts on them. Bezielle is therefore a drug that selectively targets cancer cell mitochondria, and is distinguished from other such drugs by its ability to induce not only inhibition of OXPHOS but also of glycolysis. This study provides a better understanding of the mechanism of Bezielle's cytotoxicity, and the basis of its selectivity towards cancer cells.

## Introduction

Bezielle (BZL101, FDA IND#59.521) is an extract prepared from the aerial parts of herb *Scutellaria barbata* that has anti-cancer properties. *S. barbata* extract was shown to have cytotoxic activity against various tumor cell lines *in vitro* [1], [2], [3], [4], [5], [6], [7], [8], [9], [10] and antitumor activity *in vivo* [11], [12], [13] in models of renal, hepatocellular and prostate carcinoma. More importantly, two early stage clinical trials with Bezielle showed promising efficacy and excellent safety for treatment of advanced breast cancer [14], [15]. In the phase Ia clinical trial sixteen patients, all of which went through multiple treatment therapies prior to enrollment were evaluable for response. Four patients had stable disease for >90 days (25%) and 3/16 had SD for >180 days (19%). Five patients had objective tumor regression [15]. Subsequent small scale escalation trial with an improved formulation of Bezielle showed a similarly promising efficacy [14]. The need for new drugs to curb morbidity and mortality of metastatic cancers cannot be overemphasized. In particular, orally available drugs with less toxicity are urgently needed. Bezielle fulfills these unmet needs, and larger clinical studies to provide more definitive data regarding safety and efficacy are planned.

We have reported previously that Bezielle kills selectively tumor cells while sparing non-transformed cells *in vitro* [8], [15]. This selectivity might account for its superior safety profile demonstrated in early clinical trials when compared to other approved cytotoxic agents. To date, analyses of the cellular effects of Bezielle have revealed that the basis for Bezielle's selectivity is the induction of high levels of reactive oxygen species (ROS) in tumor cells that is strongly attenuated in normal cells. ROS induced by Bezielle cause extensive DNA damage and hyper activation of PARP-1 in tumor cells. Significantly, Bezielle induced a modest DNA damage in non-transformed cells that was repaired and did not involve a hyper activation of PARP [8]. In tumor cells, activation of PARP depleted NAD+ and ATP that are used to synthesize poly-ADP ribose for PAR-ylation of a number of cellular proteins involved in DNA damage repair, transcriptional control and regulation of cell cycle [16], [17], [18]. Depletion of cytosolic NAD+ is most likely the cause of the subsequent selective inhibition of glycolysis [19], [20], [21] that is observed in Bezielle treated tumor but not in normal cells [8]. Inhibition of glycolysis as well as collapse of redox status (manifested in depletion of NADPH and GSH in tumor cells treated by Bezielle) are the likely cause of cell death triggered by Bezielle. Indeed, our results show that Bezielle induces mainly necrotic death [8] that is known to be associated with the hyperactivation of PARP-1 [21], [22], [23]. A recent detailed combined proteomic and metabolomic analysis of breast cancer cells treated with Bezielle revealed induction of oxidative stress damage followed by redistribution of metabolic fluxes [24]. Specifically, three families of enzymes involved in maintenance of redox potential were affected by Bezielle: glutathione- and thioredoxin-related proteins and peroxiredoxins. The largest subset of proteins affected by Bezielle were those involved in metabolic pathways, including glycolysis, Krebs cycle and fatty acid metabolism. These data support the hypothesis that Bezielle critically affects tumor cells metabolism through oxidative damage.

In this study, we have further analyzed the mechanisms of Bezielle induced cell death and its selectivity. We report that mitochondria are the primary cellular target of Bezielle, and the previously described cellular consequences of treatment with Bezielle are triggered through the effects of this botanical extract on mitochondria. By examining the metabolic effects of Bezielle we have found that Bezielle inhibits not only glycolysis but also oxidative phosphorylation, thus compromising both cellular energy production pathways.

## Materials and Methods

### Reagents and antibodies

Bezielle extract was prepared as described earlier for the clinical grade material [15]. Briefly, the dried raw herb was ground to a fine powder, added at a ratio 1∶10 (weight to volume) to pre-warmed water and simmered at 80°C for 30 minutes. The supernatant was separate from the insoluble solids by centrifugation at 5,000 rpm, and subject to a freeze-drying. The resulting powder was dissolved in water at 50 mg/ml and sterile filtered prior to use.

Diphenylene iodonium (DPI), N-acetyl-cystein, apocynin, FCCP (p-trifluoromethoxy carbonyl cyanide phenyl hydrazone), antimycin A and other chemicals were purchased from Sigma. Fluorescent indicators of ROS CM-H_2_DCFDA and MitoSox Red were from Molecular Probes/InVitrogen. Antibodies to PAR were from Becton-Dickinson, to NOX4 from Epitomics, to subunit 2 of the mitochondrial complex IV from Mitosciences, to LC3 from Abgent, and to SQSTM from Cell Signaling.

### Cell culture and treatments

All cell lines were obtained from the ATCC. MDAMB231 were propagated in RPMI with 10% FCS. MCF10A were propagated in DME/F12 with 5%FCS, 50 µg/ml pituitary extract, 10 µg/ml insulin, 0.5 µg/ml hydrocortisone and 0.02 µg/ml EGF (Sigma). Hs578T and SKBr3 cells were cultured in DMEM with 10% FCS. Preparation of the aqueous extract of Bezielle was described earlier [8]. Cells were treated with Bezielle at 250 µg/ml (dry weight per volume), unless otherwise indicated in the Legends to Figures, or with water (labeled as “untreated” throughout the paper). All treatments with Bezielle were done in full growth media lacking pyruvate.

### Measurement of the ROS

Fresh stocks of ROS indicators CM-H_2_DCFDA and MitoSox Red were prepared in DMSO at a concentration of 0.5 mM and diluted into warm growth media to the concentration of 2.5 µM. The medium containing one of the indicator dyes was added to cells. After incubating the cultures with the probes for 20 minutes, cells were washed with PBS, treated with trypsin and harvested for analysis by FACS. Fluorescence from CM-H_2_DCFDA was collected on FL1 channel, and for MitoSox Red on FL2 channel. Each experiment included cells that were not subjected to treatment with Bezielle. The fluorescence levels of these untreated cells were assigned an arbitrary value of 1, and fluorescence of Bezielle treated cells is shown in Figures as “fold increase” over untreated cells.

### Lentivirus - mediated siRNA and gene expression

For NOX4 silencing, six shRNA constructs in the LKO plasmid vector were purchased from Open Biosystems/Thermo. Viruses were produced in HEK293 cells according to manufacturer's instructions and used to transduce cells, followed by selection in pre-determined concentrations of puromycin.

### Measurements of cell viability, cell numbers and apoptosis

Cell viability was determined using CCK-8 kit (Dojindo). Cell death analysis was done using the Annexin V/propidium iodide staining followed by flow cytometry. Cells were collected by trypsinization into their culture media, washed with PBS and stained with Annexin V- FITC (BioVision) and propidium iodide (PI), and analyzed immediately after a 5 minute incubation using the CellQuest software on the FACScan (Becton Dickinson). ATP levels were quantified with ATP Bioluminescence assay kit HS II (Roche Applied Science).

### Quantification of reduced glutathione using fluorescent probe monobromobimane

Cells in 96 well plates were treated as desired, the treatment media was removed and substituted with media containing 8 µM monobromobimane (mBB). Fluorescence was read on a plate reader with filters set at excitation of 360 nM and emission at 460 nM.

### Western blot analysis

Whole cell lysates were electrophoresed on SDS-PAGE and transferred to nitrocellulose membranes. Membranes were blotted with antibodies at recommended concentrations overnight at 4°C and the bound primary antibodies were detected using peroxidase-conjugated secondary antibodies. Blots were developed using SuperSignal enhanced chemiluminescence kit (Pierce) and imaged on Kodak Imager ISR2000.

### Comet Assay

Comet assays were performed using the Comet assay kit from Trevigen according to the manufacturer's instructions. Briefly, cells were harvested, washed and resuspended with PBS. The cells were combined with molten, low melting point agarose at 37°C and pipetted unto Comet slides. The agarose was allowed to solidify at 4°C for 30–40 min, and slides were immersed in cold lysis solution (Trevigen, Inc.) for 30 min at 4°C. The slides were immersed next into freshly prepared alkali solution (300 mM NaOH and 1 mM EDTA) for 20 min and subjected to electrophoresis in the same alkaline buffer at 15 V (0.5 v/cm) and 300 mA for 25 min. Slides were rinsed in water and fixed in 70% ethanol for 5 min. After air-drying, the nuclei were stained with SYBR green (Trevigen, Inc.) and viewed under a fluorescence microscope. Percentages of cells with comets were quantified by two observers blinded to the identity of the slides. Nuclei were also photographed and analyzed with the TriTek CometScore image analysis software. Olive tail moment, defined as the product of percentage DNA in the tail and displacement between the position of the mean centers of mass in the heads and tails, was determined for at least 40 cells per sample.

### Metabolic analyses with Seahorse XF96 Extracellular Flux Analyzer

Cells were plated overnight on XF96 PET 96 well plates at experimentally predetermined numbers (10×10^3^ cells per well for MDAMB231 and 12×10^3^ cells per well for MCF10A). Metabolic fluxes were analyzed on Seahorse XF96 analyzer per manufacturer instructions as previously described [25]. Basal ECAR (extracellular acidification rate, in mpH/min), OCR (oxygen consumption rates, in pMoles O^2^/min) and PPR (normalized proton production rate, in nmol H+/min) values were measured for 4 cycles. Each cycle consisted of a 3 minutes media mixing time and a 5 minutes measurements time. After measuring the basal levels of ECAR and OCR for 4 cycles, the mitochondrial uncoupler FCCP at 1 µM was automatically injected into the experimental wells in order to quantify the maximum respiration capacity, or mitochondrial reserve. Following one cycle of measurements inhibitor of mitochondrial complex III antimycin A at 5 µM was injected into the experimental wells, and another measurement cycle was performed to verify that oxygen consumption was of mitochondrial origin. Each experimental point was an average of 6 to 10 replica wells in each experiment, and experiments were repeated at least 3–4 times. Experimental data were considered acceptable only when variations in pH values between mix and measure cycles were less than 0.05–0.1 pH units. This ensured that ECAR data were not artificially altered by changes in pH values, and in fact closely paralleled changes in PPR values. Normalization of values obtained in XF96 assays was performed using quantification of cellular DNA on experimental plates with the CyQuant assay (Promega). All the XF96 data are expressed as ECAR or OCR values normalized to DNA content.

## Results

### The role of mitochondria in the induction of ROS and cell death by Bezielle

**Figure 1:**
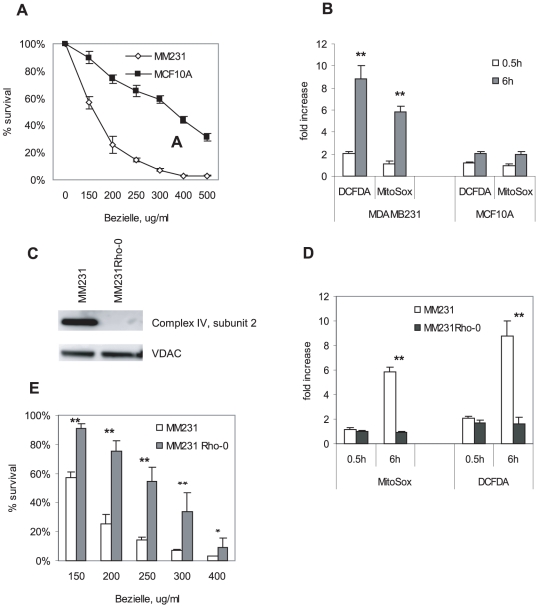
Bezielle selectively targets mitochondria of cancer cells to induce cell death. **A.** Bezielle induces cell death in tumor cells but is less cytotoxic towards non-transformed mammary cells. Breast cancer cell line MDAMB231 and non-transformed line MCF10A were treated with Bezielle at the indicated concentrations for 24 hours, and cell viability was quantified using the CCK-8 assay kit. Results are mean ± S.E. (n = 4). **B.** Cells were treated with Bezielle at 250 µg/ml for half an hour or 6 hours, loaded with the ROS sensitive probes, and analyzed by flow cytometry for green (H_2_DCFDA) or red (MitoSox) fluorescence. Results are expressed as fold increase of fluorescence in treated cells compared to untreated probe-loaded cells. Results are mean ± S.E. (n = 3). Significant differences between MDAMB231 and MCF10A are indicated (** P<0.01). **C.** Western blot analysis of MDAMBRho-0 cells selected in low concentration of ethidium bromide confirms the elimination of the mitochondrially encoded subunit 2 of the respiration complex IV, and therefore inhibition of OXPHOS. **D,** Analysis of ROS induction in Rho-0 cells performed as described in B. **E.** Analysis of cell death induction by Bezielle in Rho-0 cells performed as described in A. Significant differences between the indicated groups are indicated (*P<0.05; ** P<0.01).

We have reported previously that Bezielle is selectively cytotoxic to breast cancer cell lines but not to non-transformed cells such as immortalized mammary cells and primary fibroblasts, which are relatively resistant to its cytotoxic effect [8]. We have since tested Bezielle's cytotoxicity in over two dozens of cancer cell lines and four different immortalized cell lines and primary cells. We observed that on average the non-transformed cells are much less sensitive to Bezielle that cancer cell line (manuscript in preparation). Figure 1A illustrates these observations with two cell lines: breast carcinoma line MDAMB231 and immortalized epithelial line MCF10A. We have observed that the sensitivity to Bezielle in general does not correlate directly with the proliferation rate of the cell lines (data not shown). Thus, the proliferation rate of the non-transformed MCF10A cells is actually somewhat higher than that of cancer cell lines MDAMB231, and is much higher that that of other breast cancer cells lines that are sensitive to Bezielle [8].

The induction of death in cancer cells by Bezielle is a direct consequence of induction of ROS and oxidative stress, because co-treatment with antioxidants N-acetyl-cystein (NAC) or pyruvate inhibited DNA damage and cell death [8]. We aimed to identify the cellular sources of the Bezielle-induced ROS. Cell lines were treated with Bezielle for different times and loaded with cell-permeable indicators of ROS CM-H_2_DCFDA that becomes fluorescent in response to peroxide-type oxygen radicals [26], or MitoSox Red, highly selective for mitochondrial superoxide [27]. We have observed a gradual time dependent increase in ROS in breast cancer cell lines MDAMB231 and SKBr3, but not in MCF10A cells after treatment with Bezielle (Figure S1). Figure 1B illustrates ROS levels at 30 minutes and 6 hours after treatment. Data are expressed as fold increase of fluorescence over that seen in untreated cells loaded with the fluorescent indicator. MDAMB231 produced more ROS than MCF10A cells already after the first 30 minutes of incubation with Bezielle, but with time the difference in ROS levels induced in the two cell line became increasingly more pronounced. At six hours of treatment both peroxide and mitochondrial superoxide were increased by approximately five and six-fold, respectively, in MDAMB231 cells. The increase in ROS levels in MCF10A was much more modest, and barely exceeded two fold over the basal ROS levels even after 6 hours of treatment (Figure 1B). It is possible that the continuous accumulation of ROS in tumor cell lines is directly relevant to their sensitivity to Bezielle.

These results posed a question regarding the nature of peroxide type radicals induced by Bezielle: namely, does Bezielle induce both peroxide and superoxide, or is peroxide merely a conversion product of mitochondrial superoxide? Considering that superoxide is a short lived species of reactive oxygen, the high levels of it observed at 6 hours are likely due to continuous generation by mitochondria. The correspondingly high levels of peroxide type ROS could be resulting from the conversion of superoxide to peroxide by superoxide dismutase.

To examine the role of mitochondrial superoxide in cell death induced by Bezielle, we have employed a well-tested method of disabling mitochondrial respiration by culturing cells in presence of ethidium bromide. After culture of cells in low concentration of ethidium bromide for 6 weeks, these cells (MDAMB231Rho-0) have lost expression of mitochondrial proteins encoded in mitochondrial genome as seen in Figure 1C, rendering mitochondria not functional. This was also confirmed by examining mitochondrial oxygen consumption of MDAMB231 Rho-0 cells, which was greatly reduced (see below, Fig. 6). The Rho-0 cells were treated with Bezielle, and examined for induction of ROS and cell death. Figure 1D shows that the MDAMB231 Rho-0 cells, as expected, do not generate mitochondrial superoxide in response to Bezielle, confirming the role of mitochondrial electron transport in the induction of ROS by Bezielle. Levels of peroxide generated in Bezielle treated Rho-0 cells were somewhat increased at 30 minutes of incubation, but showed no further increase over time (Figure 1D), strongly supporting our suggestion that high peroxide levels could be due largely to conversion of mitochondrial superoxide. Most importantly, the MDAMB231 Rho-0 cells were very strongly protected from cell death induced by Bezielle (Figure 1E) suggesting the pivotal role of mitochondria in death of tumor cells treated with Bezielle.

### Potential other cellular sources of ROS induced by Bezielle

**Figure 2:**
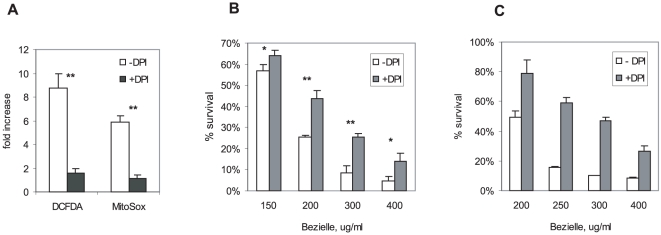
NOX inhibitor DPI inhibits induction of ROS and cell death by Bezielle. **A.** MDAMB231 cells were incubated with Bezielle in absence or presence of 0.75 µM DPI for 6 hours and examined for ROS production. **B.** Cell death in MDAMB231cells treated with Bezielle for 24 hours in absence or presence of 0.75 µM DPI. All results in A and B are mean ± S.E. (n = 3). Significant difference (*P<0.05; ** P<0.01) between the indicated groups are shown. **C**, Cell death in breast carcinoma cells SKBr3 treated with Bezielle for 24 hours in absence or presence of 0.75 µM DPI. Results are average of two experiments.

The data described above heavily implicate the mitochondrially generated superoxide in Bezielle cytotoxicity. However, while induction of mitochondrial superoxide by Bezielle was entirely prevented in Rho-0 cells, the CM-H_2_DCFDA detectable peroxide type ROS, as mentioned above, were not completely abolished (Figure 1D). We have considered the possibility that some other cellular enzymes capable of producing peroxide or extra-mitochondrial superoxide may contribute to the oxidative stress caused by Bezielle. We have used inhibitors of several cellular enzymes that produce ROS: diphenylene iodonium (DPI) and apocynin for inhibition of NADPH oxidases; allopurinol to inhibit xantine oxidase, nordihydroguaiaretic acid (NDGA) to inhibit LOX, and Nitro-L-arginine methyl ester (L-NAME) to inhibit nitric oxide synthase. MDAMB231 cells were treated with Bezielle in presence of each of these inhibitors, and the extent of cell death as well as ROS induction was quantified in relevant assays. None of the inhibitors reduced ROS levels in Bezielle-treated cells with the exception of DPI (not shown and Figure 2A). Similarly, with the exception of DPI, none showed any protective effects on cell death (not shown).

Figure 2A shows inhibition of both peroxide type ROS and mitochondrial superoxide production by DPI in Bezielle-treated cells. The observed strong inhibition of mitochondrial superoxide by DPI was unexpected, since inhibition of NADPH oxidases (NOX) should not have a profound effect on mitochondria, even though some published studies suggest the existence of a cross-talk between Nox activation and mitochondrial production of superoxide [28], [29]. However, DPI was also reported to be a potent inhibitor of mitochondrial superoxide production probably through the non-specific inhibition of NADH-ubiquinone oxidoreductase in mitochondrial complex I [30].

Not surprisingly, inhibition of Bezielle induced ROS production by DPI had a protective effect on Bezielle-induced cell death (Figure 2B). Even though DPI alone was somewhat cytotoxic to cells, it strongly attenuated cell death induced by Bezielle (Figure 1E). To rule out the possibility that the effects of DPI treatment are a particular feature of MDAMB231 cells, we have examined effects of DPI on cell death in an additional breast carcinoma cell line SKBr3. The SKBr3 cells showed an even stronger protection by DPI than MDAMB231, indicating that the protective effect of DPI towards Bezielle is not an isolated phenomenon (Figure 2C).

We have therefore examined if the non-phagocytic NOX known to be expressed in breast tumors [29], [31] could be involved in Bezielle cytotoxicity. The results of these experiments are shown in Figure S2. Briefly, even though NOX4 appears to be upregulated by Bezielle in tumor cells, partial silencing of its expression in MDAMB231 cells indicated that NOX4 produced ROS do not play a significant role in cell death induced by Bezielle.

The question, however, still remained about the mechanism of the mitochondrial ROS reduction by DPI. We have found that DPI, even at the low concentration used here, inhibits mitochondrial respiration (see below), and thus, in effect, acts as inhibitor of OXPHOS, similar to the ablation of mitochondrial function in Rho-0 cells.

### Induction of DNA damage

**Figure 3:**
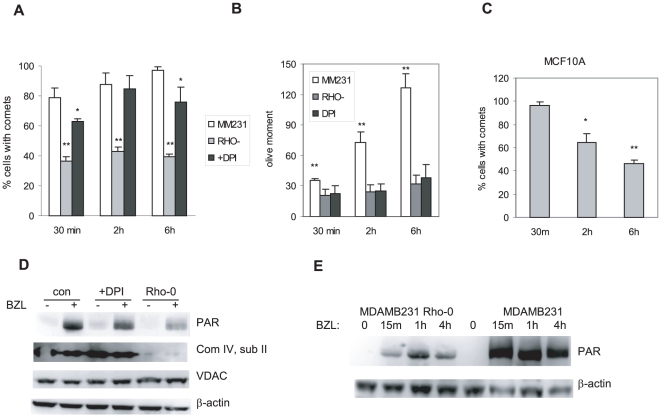
Bezielle induced DNA damage is alleviated in cells lacking functional mitochondria. **A.** MDAMB231 control cells (MM231), MDAMB231 Rho-0 variant (RHO-), or MDAMB231 cells in presence of DPI (+DPI) were treated with Bezielle for the times indicated and subjected to alkaline Comet assay analysis. Results are shown as percentages of cells forming comets, mean ± S.E. (n = 3). Significant differences (*P<0.05; ** P<0.01) between the Rho-0 and control groups, and between DPI-treated and control groups are shown. **B.** Same experiments as in A, but the results shown are mean olive moments of the comets induced by Bezielle in three cell populations. At least 50 comets were analyzed per each time point in four separate experiments. Significant differences (**P<0.01) were observed between control cells and both Rho- and DPI-treated groups. **C.** Percentages of MCF10A cells showing DNA damage at different times after incubation with Bezielle, results are mean ± S.E. (n = 3). Significant differences (*P<0.05; ** P<0.01) between cells treated for 30 minutes compared to both 2 and 6 hours are shown. **D.** Western blot analysis of PARP activation (PAR) in the indicated cells treated with Bezielle for 1 hour. Also shown are the lack of expression of the mitochondrially encoded protein (complex IV subunit II) in Rho-0 cells, expression of mitochondrial protein VDAC encoded in nuclear DNA, as well as β-actin as a loading control. **E.** MDAMB231 cells and MDAMB231-Rho-0 cells were treated with Bezielle for the indicated times and analyzed for presence of poly-ADP-ribosylated proteins. β-actin serves as a loading control.

Bezielle induces oxidative DNA damage that is critical for its ability to selectively kill cancer cells [8]. We have examined how lack of functional mitochondria and treatment with DPI affect induction of DNA damage by Bezielle in MDAMB231 cells. Cells treated with Bezielle were analyzed by Comet assay for two parameters of DNA damage: percent of cells with damaged DNA, and the olive moment (reflecting the extent of DNA damage in individual cells). As seen in Figure 3A, treatment with Bezielle led to progressively increasing numbers of cells with DNA damage for up to 6 hours. After that time point, dead cells with partially degraded or severely damaged DNA started accumulating, making the comet quantification difficult. Analysis of the comet tail moment (Figure 3B) revealed a steep increase of DNA damage per cell induced by Bezielle over time. The increasing amount of DNA damage per cell in a time-dependent manner correlates well with the observed increase in ROS levels (Figure 2B).

In MDAMB231 Rho-0 cells, less than half of the cells in the treated population showed the presence of DNA damage even after 6 hours (Figure 3A), and olive moment analysis detected low levels of DNA damage per cell that did not increase over time (Figure 3B). Presence of DPI had a limited effect on the number of cells with DNA damage, but had a strong inhibitory effect on the increase in DNA damage per cell over time. The weak effect of DPI on the number of cells with DNA damage might be related to the weaker protective effect of DPI against Bezielle induced death compared to protection in Rho-0 cells (Figure 1E and 2B).

We have also analyzed Bezielle induced DNA damage in MCF10A cells, and have observed that even in the continuous presence of Bezielle in the growth media, DNA damage in these cells was gradually reduced over time (Figure 3C). We suggest that much lower levels of ROS induced in MCF10A cells (Figure 1B) led to limited levels of DNA damage which was successfully repaired. This could explain the time dependent reduction in the DNA damage in MCF10A cells.

We have documented hyper activation of PARP in tumor cells treated with Bezielle, and its role in the induction of cell death. We have examined if PARP was activated in MDAMB231 Rho-0 cells or in DPI-treated cells. As shown in Figure 3D, PARP was strongly activated in MDAMB231 cells. In Rho-0 cells, PARP activation was significantly less robust. In DPI-treated cells, Bezielle activated PARP to levels in between these observed in control versus Rho-0 cells (Figure 3D). PAR-ylation of proteins in MDAMB231 cells continued for hours after the start of treatment with Bezielle, but in Rho-0 cells the activation of PARP was not only much weaker, but also transient in nature (Figure 3E). Altogether, the data above show that lack of functional mitochondria strongly inhibited induction of ROS, DNA damage, activation of PARP and cell death by Bezielle. We therefore conclude that mitochondria are the primary cellular target of Bezielle.

### Cancer cells treated with Bezielle undergo a collapse of oxidative defense system

**Figure 4:**
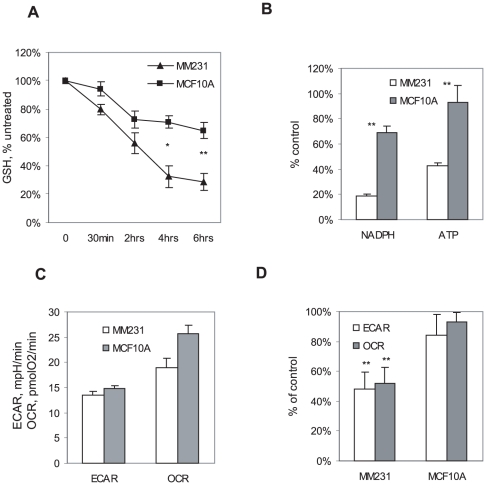
Bezielle disrupts redox and energy status in breast cancer cells. **A.** Bezielle depletes cellular store of reduced glutathione. Levels of GSH were monitored in cells using monobromobimane as described in Materials and Methods. The basal levels of GSH in untreated MDAMB231 cells were 32±11% higher than in MCF10A cells. **B.** Levels of NADPH and ATP in cells treated with Bezielle for 4 hours. Results in **A** and **B** are mean ± S.E. (n = 3). Significant differences (* P<0.05, ** P<0.01) between MCF10A and MDAMB231 are shown. **C.** Basal levels of OXPHOS (OCR) and glycolysis (ECAR) in untreated MDAMB231 cells and MCF10A cells. OCR values are in pMoles O2/min, and ECAR values are in mpH/min. Extracellular flux was analyzed using XF96 as described in Materials and Methods. Results are mean and S.E. of five experiments. **D.** Analysis of changes in OCR and ECAR in cells treated with Bezielle for 4 hours. Results are mean and S.E. of five experiments. Significant differences (** P<0.01) between Bezielle treated and untreated cells are indicated.

Previously published analysis of the transcriptional changes induced by Bezielle in tumor cells [8] indicated that the glutathione redox system is engaged in the cellular response to Bezielle, a conclusion supported by the recent proteomic and metabolomic analyses [24]. Thus, elevated expression of cystathionine-beta-synthase and glutamate cysteine ligase modifier GCLM were observed in Bezielle treated tumor cells but not in non-transformed cells. Therefore, we examined levels of reduced glutathione (GSH) in cells treated with Bezielle. The results in Figure 4A show a strong and rapid decline of GSH in MDAMB231 cells. Levels of GSH were somewhat reduced in MCF10A, but the decline in GSH was relatively mild and not sustained. At longer incubation times, GSH was almost entirely depleted in MDAMB231 cells (not shown). GSH is an important antioxidant, and conversion of oxidized GSH (GSSG) to GSH requires NADPH. We have therefore examined levels of NADPH in cells treated with Bezielle. Even after only 4 hours (Figure 4B) there was a profound depletion of NADPH in MDAMB231 cells. Evidently, the redox balance in MDAMB231 cells, but not MCF10A cells treated with Bezielle was undergoing a rapid collapse. As has already been reported by us [8], levels of ATP were also quickly depleted in MDAMB231 cells (Fig. 4B). Rapid loss of redox potential and energy is likely to play a major role in the selective cytotoxicity of Bezielle towards tumor cells supporting active OXPHOS.

### Metabolic effects of Bezielle

We have described earlier a strong and selective inhibitory effect of Bezielle on glycolysis in tumor cells, reflected in a strong reduction of lactate production and activity of glycolytic enzymes glyceraldehyde 3-phosphate dehydrogenase (GAPDH) and lactate dehydrogenase [8]. Considering the data described above that implicate mitochondria as primary target of Bezielle, we have now examined the effect of Bezielle on mitochondrial OXPHOS. We used the extracellular analyzer of metabolic fluxes XF9 (Seahorse BioSciences), which monitors glycolysis and mitochondrial OXPHOS in real time and in a non-invasive manner.

Figure 4C shows the basal rates of mitochondrial respiration (or oxygen consumption rate, OCR) and glycolysis (extracellular acidification rate, ECAR, a measure of lactate production) in MDAMB231 cells and in MCF10A cells. The respiration rates were somewhat higher in MCF10A cells perhaps reflecting their higher reliance on OXPHOS for energy production. The results of metabolic flux analyses of MDAMB231 and MCF10A cells treated with Bezielle for four hours are summarized in Figure 4D. A strong inhibition of both mitochondrial respiration and glycolysis was observed in Bezielle treated MDAMB231 cells. The metabolic effects of Bezielle on MCF10A cells were relatively minor, even though a consistent slight reduction in metabolic rates was observed. We have also examined the effects of Bezielle on proton production rate (PPR), a measure of glycolytic activity available in the Seahorse instrument that calculates proton release rates rather than changes in pH. PPR under variable setting in the instrument is independent of changes in pH. Figure S3A shows that, similar to ECAR, PPR is strongly inhibited by Bezielle in MDAMB231 and Hs578T tumor cells, but not significantly in MCF10A. We conclude that Bezielle inhibits both cellular energy-producing pathways selectively in tumor cells but not in non-transformed cells.

**Figure 5:**
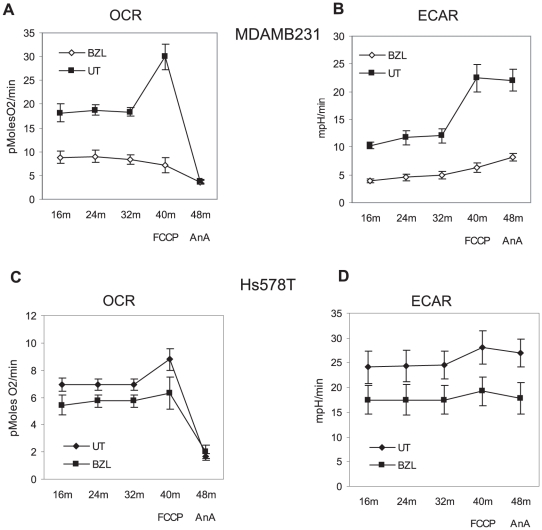
Bezielle suppresses both energy producing pathways in breast cancer cells. **A.** Oxygen consumption (OCR, (pMoles O^2^/min)) and reserve capacity in MDAMB231 cells treated with Bezielle at 300 µg/ml for four hours or left untreated. After measuring ECAR and OCR for four cycles (of which last three are shown), 1 µM FCCP was injected for one measurement cycle followed by injection of 5 µM of antimycin A and another mix-measure cycle. **B**, ECAR (mpH/min) measurements in same experiments as described in **A.** In all experiments quantifying metabolic flux each experimental point is a mean± S.E. of normalized values measured in 8 to 10 replica wells. Results in **A** and **B** are mean ± S.E. of three independent experiments. **C** and **D.** Effect of Bezielle on metabolism of breast cancer cell line Hs578T. Cells were treated with Bezielle at 300 µg/ml for 5 hours and analyzed in the Seahorse XF96 for mitochondrial respiration (OCR) and glycolytic activity (ECAR) as described above for MDAMB231 cells.

We have next examined how treatment with Bezielle affects the mitochondrial reserve capacity in MDAMB231 cells. Reserve capacity is a measure of mitochondrial energy reserve [32]. Irreversible depletion of mitochondrial reserve capacity would lead to cell death [33]. Metabolic fluxes were measured in untreated and Bezielle treated MDAMB231 cells before and after injection of the mitochondrial uncoupler FCCP into XF96 assay media followed by the inhibitor of the respiration complex III antimycin A. By uncoupling mitochondrial respiration from ATP synthesis, addition of FCCP allows the measurement of the mitochondrial reserve capacity. As seen in Figure 5A, in untreated MDAMB231 cells oxygen consumption is significantly increased after injection of FCCP, indicating that these cells could increase their mitochondrial respiration if conditions demand so. Following injection of complex III inhibitor antimycin, cell respiration is inhibited, confirming that the observed increase of OCR after FCCP addition is a true indicator of mitochondrial ability to respire above the levels needed to support energy demand. Cells treated with Bezielle for 4 hours fail to respond to FCCP by an increase in respiration altogether, which shows that the mitochondrial reserves are depleted. Treatment of healthy cells with FCCP triggers a fast increase in glycolysis (measured as either ECAR or PPR) to compensate for the failure of the mitochondrial energy production, and is observed in untreated MDAMB231 cells (Figure 4B and Figure S3B). However, in Bezielle treated cells glycolysis is already adversely affected, and shows only a minor increase in response to FCCP. Same experiments performed with MCF10A showed a nearly complete lack of an effect of Bezielle on mitochondrial reserve capacity in these cells (not shown).

Metabolic effects of Bezielle were also examined in Hs578T cells. Analysis of these cells showed that they are significantly more glycolytic than MDAMB231 cells (Figure S3A). The proton production rate (PPR) in untreated Hs578T cells was much higher than in MDAMB231 cells, demonstrating a strong preference for glycolysis in Hs578T (Figure S3A). Nevertheless, Bezielle had similar effect on metabolism of Hs578T cells, i. e., reduction in both OXPHOS and glycolytic activity (Figure 5C and 5D).

**Figure 6:**
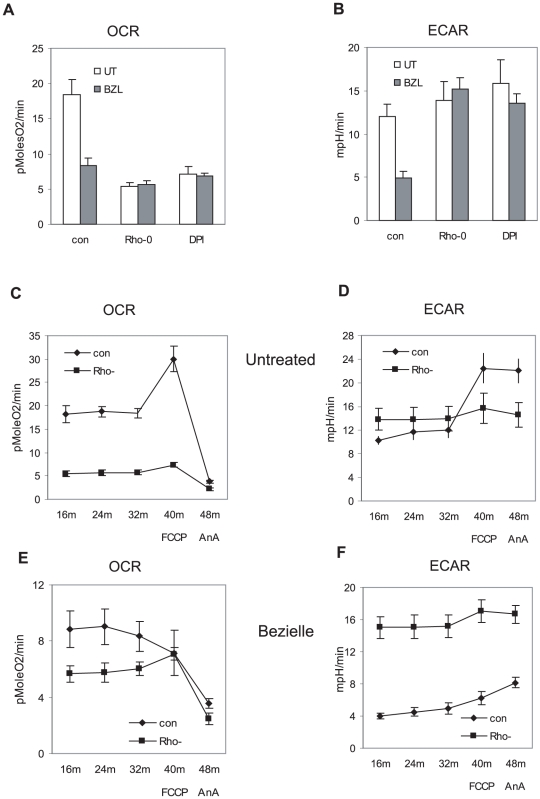
Bezielle inhibits energy production in MDAMB231 cells and depletes their mitochondrial reserve capacity only in presence of respiring mitochondria. **A.** MDAMB231 cell (con) in absence and presence of DPI, and Rho-0 variant cells were treated with Bezielle or water (UT) for 4 hours. The basal levels of OCR were measured in XF96 analyzer. **B.** Same as in A, for glycolytic activity, ECAR. Results are mean ± S.E. (n = 3). **C.** Respiration and mitochondrial reserve capacity (OCR, pMoles O^2^/min) in untreated MDAMB231 and Rho-0 cells. **D.** Glycolytic activity ECAR, (mpH/min) in untreated MDAMB231 and Rho-0 cells. **E** and **F.** OCR and ECAR respectively, in same cells treated with Bezielle for four hours. Shown are results of a representative experiment from three experiments performed.

To obtain further support for the hypothesis that mitochondria are the primary target of Bezielle, in particular as related to the observed inhibition of glycolysis, we monitored the bioenergetic changes induced by Bezielle in the Rho-0 variant of MDAMB231 cells, as well as in cells treated with DPI. As shown above, both are quite resistant to the cytotoxic effects of Bezielle (Figure 1E and 2B). The basal rate of oxygen consumption (OCR) was predictably low in Rho-0 cells, and almost as low in cells acutely treated with DPI (Figure 6A). It is most likely, therefore, that the resistance of DPI-treated cells to Bezielle could be related to the DPI-induced inhibition of mitochondrial respiration, in essence mimicking the absence of respiring mitochondria in Rho-0 cells. The already low residual respiration levels in the Rho-0 and DPI-treated cells were not further affected by Bezielle, whereas a strong inhibition of OCR was seen in Bezielle-treated control MDAMB231 cells (Figure 6A).

Examination of glycolysis showed a modest increase in untreated Rho-0 and DPI-treated cells, probably due to the compensatory cellular response of cells where OXPHOS is inhibited [34]. The glycolytic rate in control MDAMB231 cells was reduced by about half after 4 hours of treatment with Bezielle, but glycolysis was not significantly affected by Bezielle in Rho-0 or DPI treated cells (Figure 6B). These results further confirm that the lack of mitochondrial respiration protects cells from the metabolism-suppressing effects of Bezielle, strongly supporting the hypothesis that mitochondria are the primary target of Bezielle.

Next, we examined the bioenergetic effects of Bezielle on the mitochondrial reserve capacity in cells with disabled mitochondrial respiration. Figures 6C and 6D show the results of these experiments for untreated Rho-0 versus control cells (DPI-treated cells are not shown because they behaved very similar to Rho-0 cells). Treatment with the uncoupler FCCP predictably had no significant effect on OCR or ECAR in Rho-cells (Figure 6C, D). Obviously, there is no mitochondrial reserve capacity that could be revealed by uncoupling respiration in non-respiring cells; and, because FCCP had no effect on OCR in Rho-0 cells, there was no feedback effect of FCCP on glycolysis in these cells.

The effect of Bezielle on low basal levels of OCR on Rho-0 cells was very weak, and FCCP addition had almost no effect (Figure 6E), similar to untreated Rho-0 cells (Fig. 6C). This was in a marked contrast to control cells, in which OXPHOS was inhibited by Bezielle, and mitochondrial reserve capacity exhausted (Figure 6E). Compared to the strong inhibition by Bezielle of glycolysis in control MDAMB231 cells (Figure 6F), the Rho-0 cells maintained virtually the same levels of glycolytic activity after Bezielle addition. It is most likely that the ability of Rho-0 cells to maintain glycolytic activity in presence of Bezielle is instrumental in their increased survival. This also strongly suggests that mitochondrial ROS are the key causative link in the chain of events induced by Bezielle, including inhibition of glycolysis.

## Discussion

**Figure 7:**
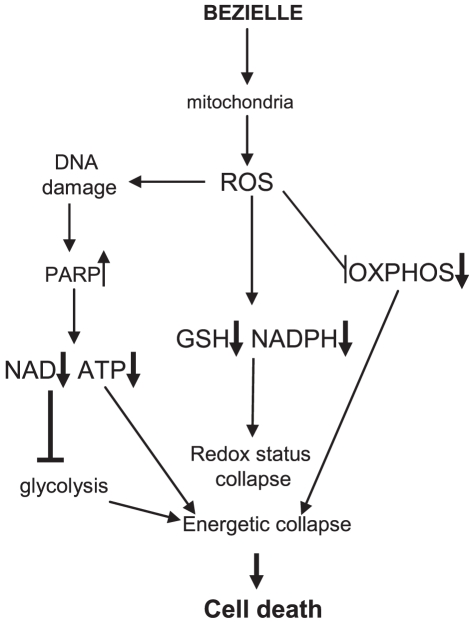
A model depicting the mechanism of action of Bezielle.

In this paper we explored the mechanism of cell death induced by Bezielle, a candidate anti-cancer botanical drug. Our previous work described the critical role of ROS and DNA damage in Bezielle-induced selective death of tumor cells. We have also documented the severe inhibition of glycolysis and energetic collapse in tumor cells treated with Bezielle. In this study we have identified the primary cellular target of Bezielle as mitochondria, and the mitochondria-produced ROS as the trigger for the subsequent events leading to the selective death of Bezielle-treated tumor cells (Figure 7). We presented the following evidence to support this conclusion: (a) generation of Bezielle-induced ROS is greatly inhibited in the non-respiring Rho-0 tumor cells lacking mitochondria or in cells where mitochondrial respiration is chemically inhibited; (b) inhibition of mitochondrial respiration suppresses DNA damage and hyper activation of PARP induced by Bezielle; (c) inhibition of mitochondrial respiration strongly attenuates inhibition of glycolysis by Bezielle (d) the Rho-0 cells are resistant to Bezielle cytotoxicity.

We have addressed the possibility that other cellular ROS-generating sources could contribute to oxidative stress induced by Bezielle treatment. In particular because Bezielle is a complex mixture of phytochemicals, we have considered the possibility that different cellular organelles and processes could be involved. Our search for other possible cellular ROS generating sources indicated that DPI is the only one of several different inhibitors examined that had a protective effect on cell death through reduction of Bezielle induced ROS. DPI is considered to be a somewhat selective inhibitor of NADPH oxidases, and we have therefore explored the role of inducible NOX4 enzyme in Bezielle induced cell death. This did not produce experimental evidence supporting the role of NOX4 in Bezielle induced cell death. Moreover, we have found, as did other investigators before us [30], [35] that DPI is not a selective inhibitor of NOX. A few studies [30], [35] warned against relying too heavily on some of the published experimental results obtained with DPI, and interpreted according to the notion that DPI is a selective inhibitor of the NOX family. We lend a strong support to these warnings here by showing directly that DPI inhibits mitochondrial respiration. In the end, it is somewhat ironic that an attempt to explore potential targets of Bezielle other than mitochondria resulted in a further confirmation that mitochondrial respiration is the primary target of Bezielle. Because disabling mitochondrial respiration led to the inhibition of the destructive cellular changes induced by Bezielle (ROS, DNA damage and inhibition of glycolysis) we have to conclude that mitochondria are the primary target of Bezielle.

Bezielle induces remarkably high levels of ROS in tumor cells that increase progressively for hours after the start of treatment. It is tempting to speculate that the progressively higher levels of ROS induced by Bezielle could be in part due to the earlier described phenomenon known as “ROS-induced ROS release” or RIRR [36], [37]. According to these publications, the initial exposure to oxidants induces production of cellular ROS that triggers the opening of mitochondrial channels followed by the collapse of the mitochondrial membrane potential and a further increased ROS generation by the electron transfer chain. Generated ROS can be released into cytosol and trigger RIRR in neighboring mitochondria. This mitochondrion-to-mitochondrion ROS-signaling leads to a further enhancement in ROS production. It is conceivable that organelles other than mitochondria could be involved in RIRR when the initially generated ROS are released into the cytoplasm.

We have examined ROS induction and cell death in tumor cells that have been treated with Bezielle for a relatively short time and transferred to fresh media without Bezielle. We observed that mitochondrial superoxide levels are progressively increasing in cells for hours after Bezielle was removed (Figure S4). The levels of ROS in these cells are not much lower than in the parallel cultures treated with Bezielle continuously. These preliminary results strongly support involvement of RIRR in the events triggered by Bezielle. Moreover our unpublished results show that a transient, 2 to 4 hour treatment with Bezielle is sufficient to induce significant death, not much lower than what was seen with a continuous treatment with Bezielle for 24 hours. This is suggestive of the possibility that the initial burst of ROS induced by Bezielle triggers cellular changes that are irreversible, and depend on secondary events such as RIRR that do not require further direct oxidative damage by Bezielle.

This study analyzed the in vitro effects of Bezielle. It should be recognized that the metabolic properties of tumor cells, ROS dynamics, and the effects of Bezielle may differ in vivo, where oxygen, pH, and nutrient conditions are different than those encountered in cell culture. In particular, it would be of interest to determine the effects of Bezielle under hypoxic conditions that are frequently encountered in tumors. The published data showing Bezielle's anticancer activity in the *in vivo* tumor models, as well as its promising efficacy in the clinical trials strongly suggest that Bezielle has an antitumor activity in vivo, though the precise mechanism of it might be somewhat different or more complex that that observed in vitro.

Our study of Bezielle suggests that much if not all of Bezielle selectivity towards tumor cells is based on the inherent differences between mitochondria of tumor versus normal cells, in particular in respect to their response to oxidative stress. This is important because if the selective cytotoxicity of Bezielle relies on the intrinsic differences between normal and tumor cells, this would make Bezielle an excellent and safe anti-cancer drug. Clearly, differential induction of ROS in tumor cells versus non-transformed cells is at the heart of the selective cytotoxicity of Bezielle. This places Bezielle in a group of drugs that selectively target mitochondria of cancer cells, and that are now known as mitocans [38]. These drugs comprise an emerging entity which generates much interest not in the least due to their selectivity. Many of them are derived from natural products [39] and are distinguished by low cytotoxicity towards normal cells. The distinguishing feature of Bezielle is that it simultaneously inhibits both OXPHOS and glycolysis. These qualities and the selectivity of Bezielle towards tumor cells are very encouraging for the further clinical development of this drug.

## Supporting Information

Figure S1 **Time dependent increase in the generation of peroxide type ROS (detected with DCFDA) in MDAMB231 and SKBr3 but not in MCF10A cells.** Cells on 96 well plates were first loaded with H2DCFDA, then incubated with Bezielle at 250 mg/ml. Fluorescence was measured at the times indicated. Data are expressed as fold increase of fluorescence in Bezielle treated cells compared to fluorescence of endogenous ROS in untreated cells. Result are representative of one of the two experiments
(PDF)

Figure S2 **A.** Expression of NOX4 is increased in MDMB231 cells treated with Bezielle. Western blot analysis of MCF10A and MDAMB231 cells treated with Bezielle for the indicated times. Cell extracts were electrophoresed and blotted with antibodies against NOX4 and GAPDH. **B.** Analysis of NOX4 expression in MDAMB231 cells transduced with a control lentivirus encoding a non-silencing sh RNA (consi) and lentivirus encoding a NOX4 shRNA. **C.** Generation of peroxide type ROS (detected with DCFDA) and mitochondrial superoxide (MitoSox) in MDAMB231 cells with partially silenced NOX4 expression. **D.** Survival of control and NOX4si cells after treatment with Bezielle. Results are average of two experiments.
(PDF)

Figure S3 **The proton production rates (PPR) undergo changes very similar to ECAR (extracellular acidification rates) in cells treated with Bezielle.** **A.** Proton production rate (PPR) is inhibited by Bezielle in tumor cell lines MDAMB231 and Hs578T, but not in MCF10A. The results show changes in PPR values in cells treated with BZL for 4 hours. Significant differences (** P<0.01) between Bezielle treated and untreated cells are indicated. **B.** Analysis of PPR in untreated MDAMB231 cells or treated as described above. The data are derived from the same metabolic flux experiments with the Seahorse XF96 for which ECAR and OCR values are shown in Figures 5A and B.
(PDF)

Figure S4 **Mitochondria of tumor cells continue to generate superoxide hours after Bezielle is removed.** MDAMB231 cells were treated with Bezielle for 1or 4 hours prior to analysis, or treated for 1 hour and then incubated in fresh medium without Bezielle for three hours (1+3 h).
(PDF)
