# Supplementary material for: Bezielle Selectively Targets Mitochondria of Cancer Cells to Inhibit Glycolysis and OXPHOS
Source: PLoS One. 2012 Feb 3;7(2):e30300. doi: 10.1371/journal.pone.0030300 (PMC3272024; doi:10.1371/journal.pone.0030300)
Supplement: Figure S1 — Time dependent increase in the generation of peroxide type ROS (detected with DCFDA) in MDAMB231 and SKBr3 but not in MCF10A cells. Cells on 96 well plates were first loaded with H2DCFDA, then incubated with Bezielle at 250 mg/ml. Fluorescence was measured at the times indicated. Data are expressed as fold increase of fluorescence in Bezielle treated cells compared to fluorescence of endogenous ROS in untreated cells. Result are representative of one of the two experiments (PDF) [file pone.0030300.s001.pdf]

Supplemental Figures

Bezielle Selectively Targets Mitochondria of Cancer Cells to inhibit Glycolysis and OXPHOS

Vivian Chen, Richard E. Staub, Sylvia Fong, Mary Tagliaferri, Isaac Cohen and Emma Shtivelman

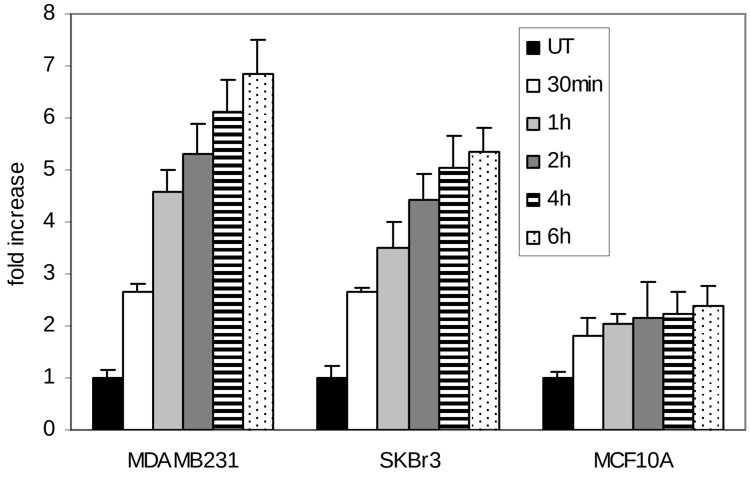

Figure S1. Time dependent increase in the generation of peroxide type ROS (detected with DCFDA) in MDAMB231 and SKBr3 but not in MCF10A cells. Cells on 96 well plates were first loaded with H_2DCFDA, then incubated with Bezielle at 250 μg/ml. Fluorescence was measured at the times indicated. Data are expressed as fold increase of fluorescence in Bezielle treated cells compared to fluorescence of endogenous ROS in untreated cells. Results are representative of one of the two experiments.
