# Supplementary material for: Bezielle Selectively Targets Mitochondria of Cancer Cells to Inhibit Glycolysis and OXPHOS
Source: PLoS One. 2012 Feb 3;7(2):e30300. doi: 10.1371/journal.pone.0030300 (PMC3272024; doi:10.1371/journal.pone.0030300)
Supplement: Figure S2 — A. Expression of NOX4 is increased in MDMB231 cells treated with Bezielle. Western blot analysis of MCF10A and MDAMB231 cells treated with Bezielle for the indicated times. Cell extracts were electrophoresed and blotted with antibodies against NOX4 and GAPDH. B. Analysis of NOX4 expression in MDAMB231 cells transduced with a control lentivirus encoding a non-silencing sh RNA (consi) and lentivirus encoding a NOX4 shRNA. C. Generation of peroxide type ROS (detected with DCFDA) and mitochondrial superoxide (MitoSox) in MDAMB231 cells with partially silenced NOX4 expression. D. Survival of control and NOX4si cells after treatment with Bezielle. Results are average of two experiments. (PDF) [file pone.0030300.s002.pdf]

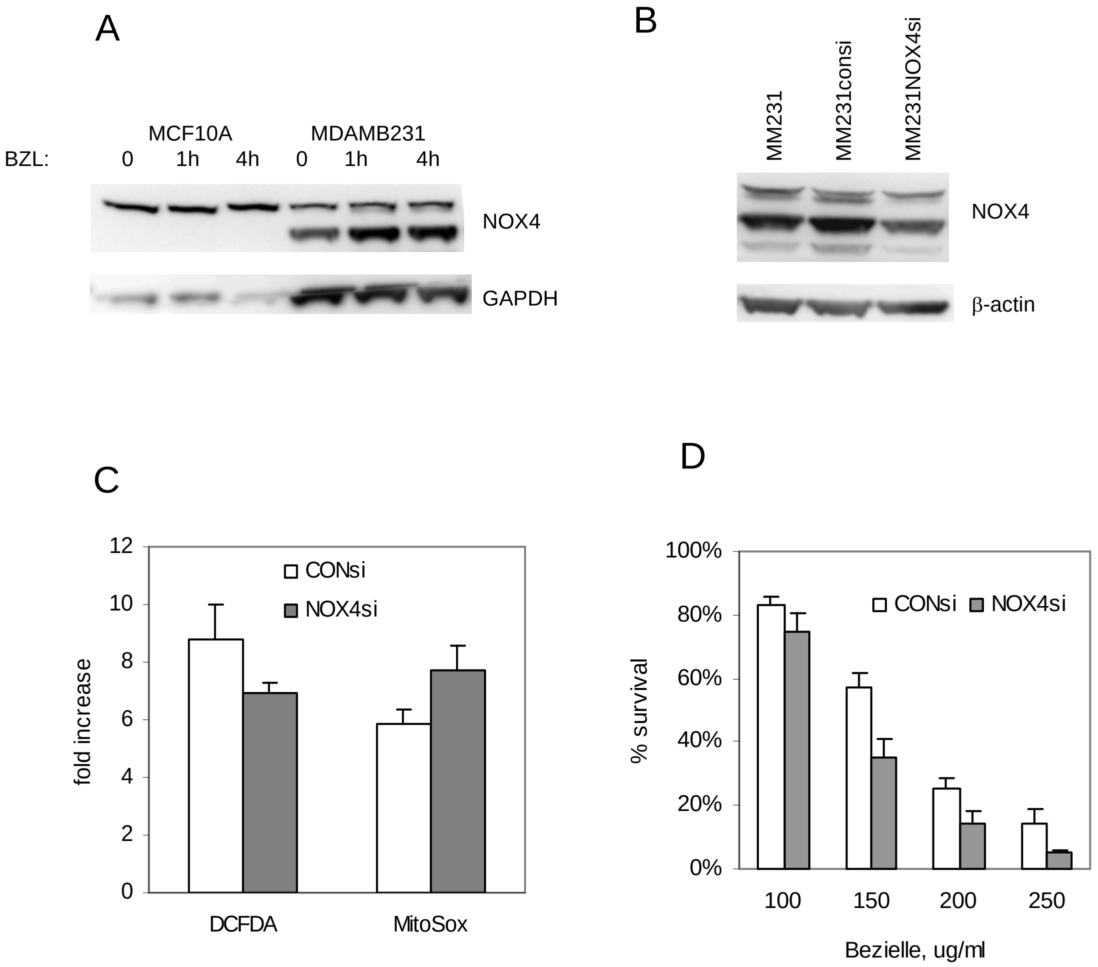


**Figure S2.** Analysis of the role of NOX4 in responses to Bezielle. **A**. Expression of NOX4 is increased in MDMB231 cells treated with Bezielle. Western blot analysis of NOX4si in MCF10A and MDAMB231 cells treated with 250 μg/ml Bezielle for the indicated times. B. Analysis of NOX4 expression in MDAMB231 cells transduced with a control lentivirus encoding a non-silencing sh RNA (consi) and lentivirus encoding a NOX4 shRNA. **C**. Generation of peroxide type ROS (detected with DCFDA) and mitochondrial superoxide (MitoSox) in MDAMB231 cells with partially silenced NOX4 expression. **D**. Survival of control and NOX4si cells after treatment with Bezielle. Results are average of two experiments.
