# Supplementary material for: Bezielle Selectively Targets Mitochondria of Cancer Cells to Inhibit Glycolysis and OXPHOS
Source: PLoS One. 2012 Feb 3;7(2):e30300. doi: 10.1371/journal.pone.0030300 (PMC3272024; doi:10.1371/journal.pone.0030300)
Supplement: Figure S3 — The proton production rates (PPR) undergo changes very similar to ECAR (extracellular acidification rates) in cells treated with Bezielle. A. Proton production rate (PPR) is inhibited by Bezielle in tumor cell lines MDAMB231 and Hs578T, but not in MCF10A. The results show changes in PPR values in cells treated with BZL for 4 hours. Significant differences (** P<0.01) between Bezielle treated and untreated cells are indicated. B. Analysis of PPR in untreated MDAMB231 cells or treated as described above. The data are derived from the same metabolic flux experiments with the Seahorse XF96 for which ECAR and OCR values are shown in Figures 5A and B. (PDF) [file pone.0030300.s003.pdf]

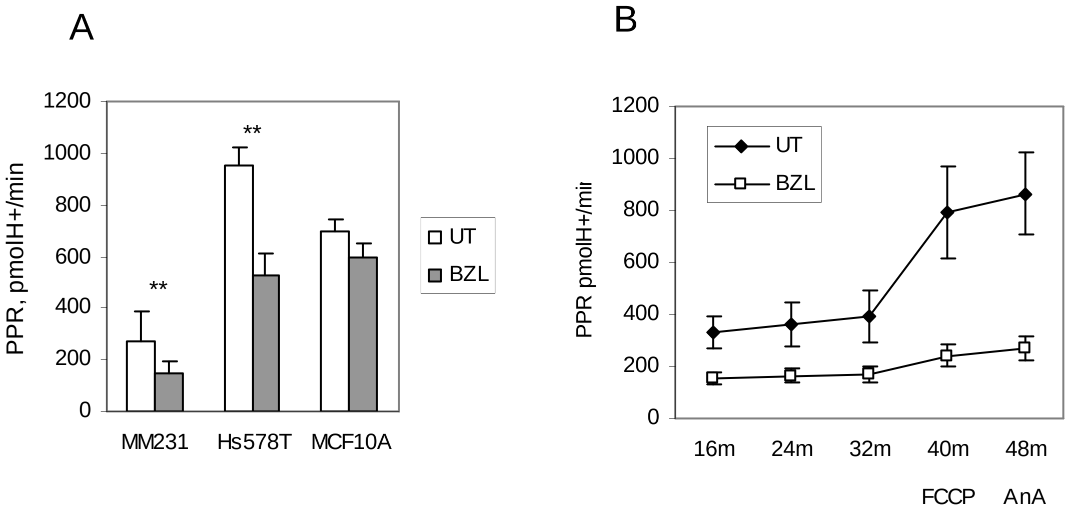

Figure S3. The proton production rates (PPR) undergo changes very similar to ECAR (extracellular acidification rates) in cells treated with Bezielle. **A**. Proton production rate (PPR) is inhibited by Bezielle in tumor cell lines MDAMB231 and Hs578T, but not in MCF10A. The results show changes in PPR values in cells treated with BZL for 4 hours. Significant differences (** P<0.01) between Bezielle treated and untreated cells are indicated. **B**. Analysis of PPR in untreated MDAMB231 cells or treated as described above. The data are derived from the same metabolic flux experiments with the Seahorse XF96 for which ECAR and OCR values are shown in Figures 5A and B.
