# Supplementary material for: Bezielle Selectively Targets Mitochondria of Cancer Cells to Inhibit Glycolysis and OXPHOS
Source: PLoS One. 2012 Feb 3;7(2):e30300. doi: 10.1371/journal.pone.0030300 (PMC3272024; doi:10.1371/journal.pone.0030300)
Supplement: Figure S4 — Mitochondria of tumor cells continue to generate superoxide hours after Bezielle is removed. MDAMB231 cells were treated with Bezielle for 1or 4 hours prior to analysis, or treated for 1 hour and then incubated in fresh medium without Bezielle for three hours (1+3 h). (PDF) [file pone.0030300.s004.pdf]

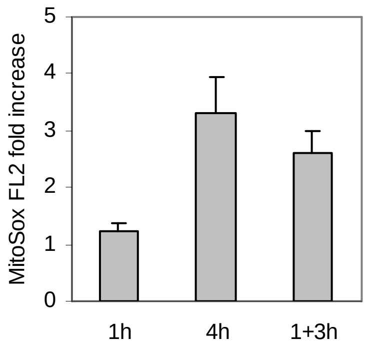

Figure S4. Mitochondria of tumor cells continue to generate superoxide hours after Bezielle is removed. MDAMB231 cells were treated with Bezielle continuously for 1 or 4 hours prior to analysis, or treated for 1 hour and then incubated in fresh medium without Bezielle for three hours (1+3h).
